# Finite element analysis of the Fibula's contribution to lower extremity torsional stiffness

**DOI:** 10.1016/j.jor.2024.10.007

**Published:** 2024-10-09

**Authors:** Angelo Alito, Andrea Vitali, Antongiulio Bruschetta, Elvira Maria Mantineo, Vincenzo Filardi

**Affiliations:** a Department of Biomedical, Dental Sciences and Morphological and Functional Images, University of Messina, 98125, Messina, Italy; b Department of Clinical and Experimental Medicine, University of Messina, 98125, Messina, Italy; c Orthopaedic Institute of Southern Italy “Franco Scalabrino”, 98165, Messina, Italy; d D.A. Scientiﬁc Research and Internationalization, University of Messina, Via Consolato Del Mare 41, 98121, Messina, Italy

**Keywords:** *CAD*, *Fibula*, *Finite element analysis*, *Lower extremity stiffness*, *Stress distribution*

## Abstract

**Aims:**

The purpose of this article is to investigate the effects of the fibula on the torsional stiffness of the lower limb. A comprehensive model of the lower limb was constructed, including the resected femur, patella, tibia, fibula, and foot, with tendons and ligaments. Two configurations were developed, with and without the presence of the fibula, to evaluate the resulting stress state and consequently determine the contribution of the fibula to the torsional stiffness of the lower limb.

**Methods:**

The finite element method was used to analyse how the fibula affects the stress distribution in the lower extremity under body weight loading and torsional forces. A detailed three-dimensional solid model of the lower extremity with and without the fibula was constructed. Loading conditions were imposed simulating an axial compressive load of 700 N applied to the upper extremity of the resected femur and a torsional load of 6000 Nmm applied to the proximal femur, and a fixed constraint was imposed on the foot.

**Results:**

Removing the fibula results in an increase in stress on all the tendons and ligaments tested. The increases ranged from 4 % (patellar tendon) to 21 % (lateral retinaculum). This suggests that the fibula plays a significant role in the distribution of mechanical stress in the lower extremity. The most affected structures are the lateral retinaculum and the posterior cruciate ligament, both with a 21 % increase in stress, suggesting that these structures may compensate more for the absence of the fibula.

**Conclusion:**

The fibula plays a critical role in maintaining the structural integrity and biomechanical function of the lower extremity. It acts as a lateral strut, contributing to the stability of the ankle and knee by providing an attachment point for several muscles and ligaments that are essential for coordinating complex movements and providing stability during dynamic activities.

## Highlights

• To study the fibula’s effect on lower limb torsional stiffness and stress distribution by using a finite element model.
• Key findings: Fibula removal increased stress on tendons/ligaments, with a 4% rise in the patellar tendon and 21% in the lateral retinaculum.
• The fibula provides stability to the lower limb, helping the ankle/knee joint coordination during movement.

## 1 Introduction

The fibula is essential in resisting torsional forces in the leg by providing lateral stabilisation, distributing forces, supporting muscles and maintaining proper alignment through its joints and ligaments.^1^ Studies have shown that it contributes significantly to axial load resistance, approximately 11–25 % in humans and that the fibula bears approximately 7 %,^2^^,^^3^ to 17 %^4^ of the compressive load in the weight-bearing lower extremity.^5^ The fibula provides also lateral support to the leg,^5^ helping to prevent torsional forces that can cause excessive leg rotation, especially during activities such as running and jumping.^6^ This bone also acts as a protective barrier, by forming the fibular tunnel, for the common peroneal nerve, a sensorimotor nerve divided into the superficial and the deep fibular nerve.^1^

The interosseous membrane (IOM) is a significant contributor to load sharing, accounting for up to 30 % of load transfer to the fibula.^7^^,^^8^ The role of the fibula in torsion is less clear and has mainly been studied in the context of ankle stability, where it plays a relatively minor role.^9^^,^^10^

The proximal and distal tibio-peroneal joints, along with the associated ligaments, work to maintain the proper alignment of the tibia and fibula, which is critical to resisting torsional forces and preventing over-rotation of the bones maintaining mobility and stability of ankle and knee.^1^ The syndesmotic ligament complex is involved in maintaining the integrity between the distal tibia and the fibula.^11^ It helps also to ensure the bones connection by resisting the axial rotational and translational forces.^12^ In particular, the distal tibiofibular syndesmosis, is fundamental for the correct dynamic function of the ankle and lower limb.^13^

Furthermore, the fibula is the site of attachment for several muscles, including the peroneal and the calf muscles which are essential for leg stability, movement, strength, and gait speed variation.^14^^,^^15^

During movements that involve rotation of the leg (e.g. walking on uneven surfaces, changing direction), the fibula works with the tibia to distribute torsional forces along the length of the leg helping to prevent injury and keeps the structural integrity of the lower limb.^16^

The primary function of the distal tibiofibular syndesmosis ligaments is maintaining ankle stability and load transmission during gait.^17^ Therefore, patients with syndesmotic lesions frequently present with ankle instability and weight-bearing difficulties, as well as ankle pain.^18^

Previous research has primarily focused on the compressive loading behaviour of the fibula, often excluding the knee and its stabilising structures.^19^ Then, these studies typically applied loads to the tibia or ankle, neglecting more physiologically relevant loading scenarios involving the femur and knee.

The purpose of this article is investigating the effects of the fibula on the torsional stiffness of the lower limb. So, a comprehensive model of the lower limb was constructed, including the resected femur, patella, tibia, fibula, and foot, together with their respective tendons and ligaments. Two configurations, with and without the fibula, were developed to evaluate the resulting stress state and consequently determine the contribution of the fibula to the torsional stiffness of the lower limb.

## 2 Methods

**Fig. 1:**
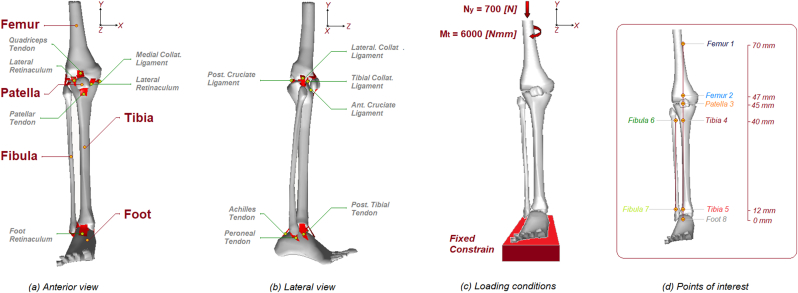
Description of the different bony and soft tissue parts of the model in anterior view (a), lateral view (b), loading conditions (c) and points of interest (d).

This reseearch used the finite element (FE) method to analyse how the fibula affects the stress distribution in the lower extremity under body weight loading and torsional forces. A detailed three-dimensional (3D) solid model of the lower extremity was constructed using CT imaging. After modelling, the 3D model was converted into an FE model, which allowed the application of loading and boundary conditions to determine the stress distribution in the tibia region. Two numerical models of the lower extremity were obtained by matching soft tissue nuclear magnetic resonance (MRI) and bone computed tomography (CT) images from a normal adult patient, the first simulating the full model and the second without the fibula. Table 1 and Fig. 1 (a) and (b) show the geometric characteristics and the geometric properties of each modelled part. Bone material characteristics were assumed to be linearly elastic, isotropic, and homogeneous, with a distinction between cortical and trabecular bone. The interfaces of contact have been determined by the definition of a penalty based method with a weight factor and a coefficient of friction of 0.4.^20^ Loading conditions were imposed simulating an axial compressive load of 700 N applied to the upper extremity of the resected femur and a torque load of 6000 Nmm applied to the proximal femur to cause rotation of the tibia and fibula. Finally, a fixed constraint was applied to the foot, see Fig. 1 (c). The direction of rotation was designed to apply an external rotation to the proximal femur, simulating the toe off phase of normal gait, where the tibia and femur rotate simultaneously and in the same direction, causing an external rotation of the foot. During this phase of gait, the knee is extended, and the foot and ankle take on the shape and resistance of a rigid lever to help perform the propulsion phase. Non-linear FE analyses were performed on the complete lower extremity model and on the second without the fibula using Abaqus version 5.4 (Hibbitt, Karlsson and Sorensen, Inc., Pawtucket, RI) with the geometric non-linearity and automatic time step options. With the aim of investigating how the different bony parts react when subjected to the combined effects of normal and torsional loading, in the presence or absence of a critical bony element such as the fibula, 8 different points were selected to evaluate the mechanical properties in terms of von Mises equivalent stress and angular displacement. The points were selected as follows: Points 1 and 2 on the proximal and distal areas of the femur, point 3 on the medial part of the patella, points 4 and 5 on the proximal and distal areas of the tibia, points 6 and 7 on the proximal and distal areas of the fibula, and finally point 8 on the anterior area of the cuboid bone of the foot.

**Table 1:** Model component geometric and mechanical properties.

geometrical properties of bony parts		
Part	Nodes	Tetra Elements
Femur	*2691*	*11325*
Patella	*685*	*2567*
Tibia	*1366*	*4816*
Fibula	*672*	*1996*
Foot	*1509*	*3254*

mechanical properties of bony parts	
**Cortical bone**	
Young Mod. = *17000 [MPa]*	Poisson ratio = *0.3*

**Trabecular bone**	
Young Mod. = *350 [MPa]*	Poisson ratio = *0.25*

geometrical properties of soft tissues		
Part	Nodes	Tetra Elements
Quadriceps Tendon	*296*	*878*
Lat. Retinaculum	*325*	*914*
Patellar Tendon	*308*	*875*
Med. Collat. Lig.	*124*	*286*
Lat. Collat. Lig.	*213*	*546*
Tibial Coll. Lig.	*269*	*682*
Ant. Cruciate Lig.	*60*	*33*
Post. Cruciate Lig.	*154*	*70*
Achilles Tendon	*121*	*289*
Peroneal Tendon	*135*	*336*
Post. Tibial Tendon	*178*	*256*
Foot Retinaculum	*223*	*635*

mechanical properties of soft tissues	
**Ligaments and tendons**	
Young Mod. = *366 [MPa]*	Poisson ratio = *0.4*

## 3 Results

**Fig. 2:**
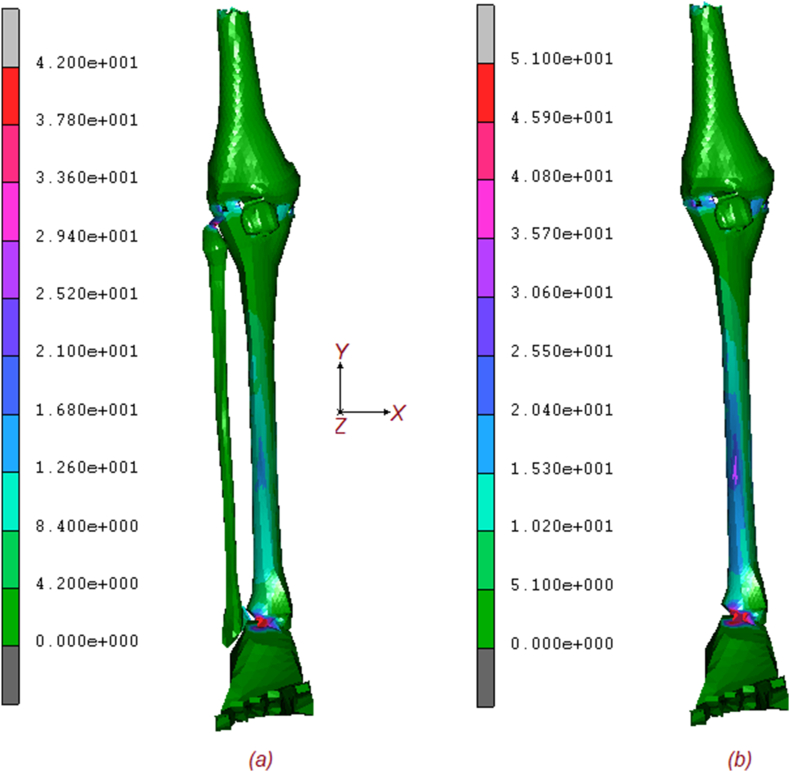
Contour maps of the equivalent von Mises stress on the two models: (a) full model; (b) model without fibula.

**Fig. 3:**
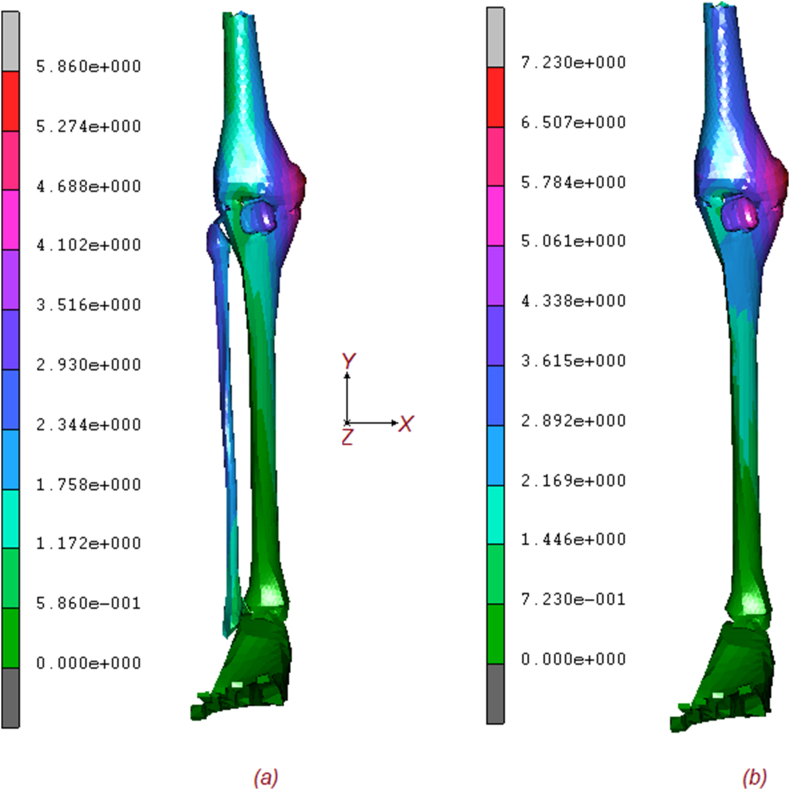
Contour maps of displacements on the two models: (a) complete model; (b) model without fibula.

**Fig. 4:**
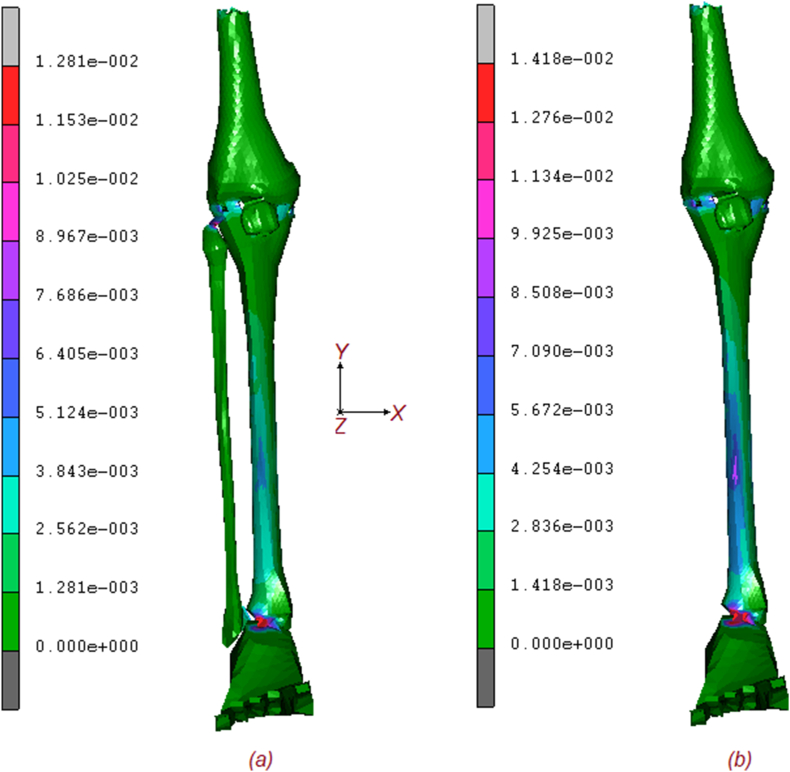
Contour maps of equivalent elastic strain on the two models: (a) complete model; (b) model without fibula.

**Fig. 5:**
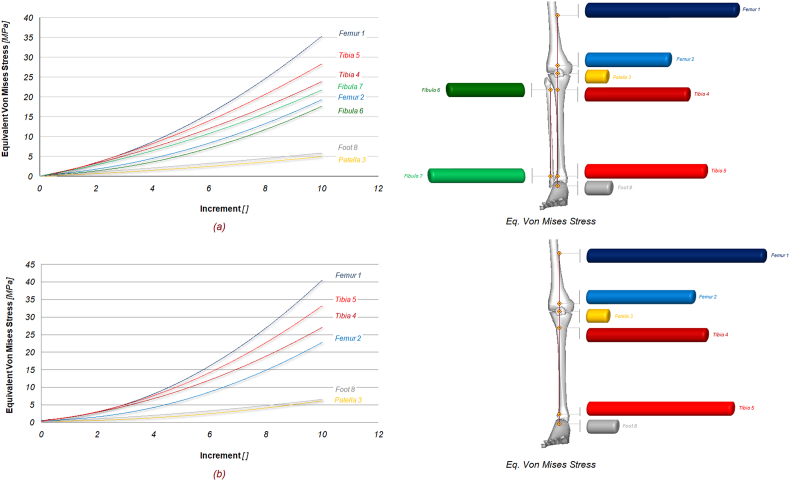
Curves of increment [ ] vs. equivalent von Mises stress (MPa) of the eight selected points in the full model (a) and in the model without fibula (b).

Two numerical models, with and without fibula, were tested to analyse the torsional stiffness of the lower extremity subjected to normal and torque loading. Fig. 2 shows the equivalent von Mises contours of stress of the two models. As it is possible to notice tibia endures an important quote of stress, but the higher amount is reached on ligaments, posterior tibial tendon. Foot retinaculum, patellar tendon, and collaterals ligaments. The maximum stress on configuration (a) is around 42 MPa, while (b) reaches values of 51 [MPa] with a stress increase of around 21 %. The effect of the fibula is obvious as its absence implies a general increase in stresses in almost every part of the lower extremity. Fig. 3 shows the comparative contour map description of the displacements for both models. Again, the maximum value of displacement for the (a) configuration is about 5.86 mm, while the (b) shows a value of 7.23 mm, for a percentage increase of about 23 %. Finally, in Fig. 4, the contour maps are related to the equivalent elastic strain, which confirms an increasing trend of the displacement for the (b) configuration, respectively 0.0128 μmm/mm for (a) and 0.0141μmm/mm for (b). Fig. 5 shows the exact position of each studied point and the relationship between the increment [/] and the equivalent von Mises stress [MPa], including the fibula (a) and excluding it (b). As can be seen by comparing the results, blue curve 1 (Femur 1) experiences the highest stress, reaching approximately 36 MPa for (a) configuration and 40 MPa for (b) configuration, at increment 10, the removal of the fibula leads to a noticeable increase in stress on Femur 1. The cyan line (Femur 2) shows stress levels in (a) configuration, around 15 MPa, indicating a significant but slightly less load-bearing role than Femur 1. Stress levels in femur point 2, in (b) configuration, increase to approximately 22 MPa, indicating additional load bearing in the absence of the fibula. The yellow line (patella 3) of the (a) configuration experiences the least stress, around 5 MPa, indicating that it bears the least load of the bony parts during the load increments. The (b) configuration still reaches the lowest stress, around 6 MPa, indicating a small increase but still the least load bearing part. The stress on patella 3 remains relatively unchanged, indicating a minimal effect of the removal of the fibula.

**Fig. 6:**
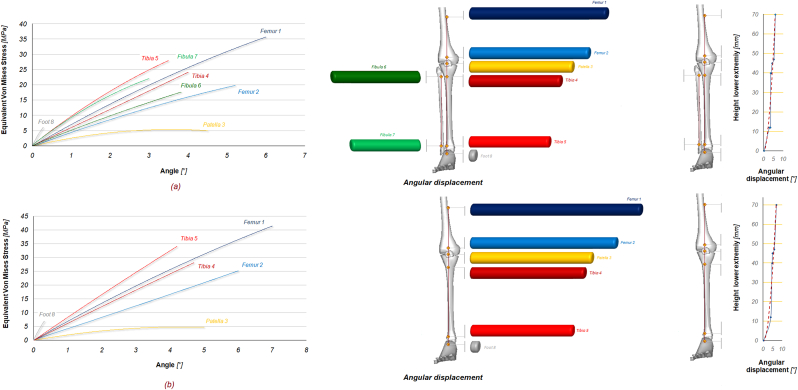
Angular displacement [°] versus equivalent von Mises stress (MPa) curves of the eight selected points in the full model (a) and in the model without fibula (b).

The green curve (tibia 4) experiences a stress that reaches about 24 MPa for the (a) configuration, indicating a significant but lower load-bearing role compared to the tibia at point 5. The stress rises to around 28 MPa at the same point in the (b) configuration, indicating a higher load-bearing role without the fibula, but it is less pronounced than in Tibia 5. The red curve (Tibia 5) shows the second highest stress, with stress levels approaching 28 MPa for the (a) configuration and 34 MPa for the (b) configuration at the same increment. This shows a significant load bearing function, Tibia 5 experiences a significant increase in stress in the absence of the fibula, suggesting that the fibula helps to distribute the load away from the tibia. The green line (fibula 6) shows stress approaching 19 MPa, which indicates a lesser, but still significant, contribution to help distribute the load. The light green line (Fibula 7) approaches 22 MPa, showing its involvement in load distribution, but less than the tibia and femur, but still able to reduce the resulting stress on them. The grey line (foot 8) experiences a relatively low stress in the (a) configuration, around 6 MPa, showing that it is less affected by load increments compared to other parts, the stress remains quite the same for the (b) configuration (7 MPa), showing a minimal effect compared to other parts. Fig. 6 shows the exact position of each point studied and the relationship between the angular rotation [°] and the equivalent von Mises stress [MPa] with the fibula (a) and without it (b). As can be seen by comparing the results, the blue curve 1 (Femur 1) experiences the highest stress, reaching about 35 MPa at about 6°, for the (a) configuration, while in (b) it is about 40 MPa at about 7°. Removal of the fibula increases the stress on femur 1, especially at higher angular displacements. The cyan line (Femur 2) shows stress levels in the (a) configuration of around 15 MPa at around 5°, indicating a significant but slightly lower load bearing role than Femur 1. Stress levels in (b) increase to approximately 25 MPa at 6°, indicating additional load bearing in the absence of the fibula. The yellow line (patella 3) of the (a) configuration experiences the least stress, around 4.5 MPa at around 5°, indicating that it is the least load bearing of the bony parts during load increments, whereas in (b) it reaches 5 MPa at 5°. The patella experiences the least stress, indicating that it is the least load bearing part during angular rotation. The dark red curve (tibia 4) experiences stress that reaches approximately 24 MPa at 4° for (a) and 28 MPa at 4.7° for (b), indicating a significant but lower load bearing role compared to the tibia at point 5. The stress rises to approximately 28 MPa at the same point in the (b) configuration, indicating a substantial load-bearing role, but slightly less than the tibia in part 5, and that the absence of the fibula increases the load on the tibia. The red curve (tibia 5) shows the second highest stress, with stress levels approaching 28 MPa at 3.5° for (a) and 34 MPa at 4.2° for (b), showing a significant increase in stress levels without the fibula, highlighting its role in reducing stress on the tibia. Points on the fibula, Fibula 6 (green line) and Fibula 7 (light green line), show stress levels of 18 MPa at 3.8° and 22 MPa at 3° respectively, showing that the fibula contributes significantly to stress management, but less so than the tibia and femur. Finally, foot 8 has a relatively low stress in both configurations, suggesting that it is less affected by angular rotation compared to other parts, for (a) it is 6 MPa at 0.3° and 7 MPa at 0.35° in the other configuration. The femur consistently shows the highest stress in both models, highlighting its primary load bearing function, the tibia also bears a significant amount of stress, with notable increases when the fibula is absent, highlighting the collaborative load bearing role of the tibia and fibula. Table 2 shows the equivalent von Mises stress values for tendons and ligaments, comparing (a) and (b) configurations, and the percentage difference in stress values between the two conditions. Removal of the fibula from the lower extremity results in an increase in stress for all tendons and ligaments measured. The increases range from 4 % (patellar tendon) to 21 % (lateral retinaculum). The most affected structures are the lateral retinaculum and the posterior cruciate ligament, both with a 21 % increase in stress, suggesting that these structures may compensate more for the absence of the fibula. The quadriceps tendon, tibial collateral ligament, Achilles’ tendon and foot retinaculum show moderate increases (13–15 %). This indicates a noticeable but not overwhelming reliance on the fibula for load distribution. Conversely, the patellar tendon shows the least change, suggesting that it is more independent of the fibula in distributing its mechanical load.

**Table 2:** Eq. Von Mises stress values for tendons and ligaments, comparing (a) and (b) configurations.

Tendons and Ligaments	Complete lower extremity	Low. extremity without fibula	Difference
	Eq. V. Mises Stress *[MPa]*	Eq. V. Mises Stress *[MPa]*	
Quadriceps tendon	*22*	*25*	*14 %*
Lat. Retinaculum	*42*	*51*	*21 %*
Medial Coll. Lig.	*34*	*40*	*18 %*
Patellar Tendon	*25*	*26*	*4 %*
Lat. Coll. Lig.	*40*	*47*	*18 %*
Tibial Coll. Lig.	*35*	*40*	*14 %*
Ant. Cruciate Lig.	*30*	*36*	*20 %*
Post. Cruciate Lig.	*41*	*49*	*20 %*
Peroneal Tendon	*26*	*30*	*15 %*
Achilles tendon	*32*	*36*	*13 %*
Post. tibial tendon	*42*	*49*	*17 %*
Foot retinaculum	*24*	*27*	*13 %*

## 4 Discussion

The aim of this study was to study the effect of the fibula on the torsional stiffness of the lower limb. For this study, a detailed model of the lower limb was constructed, including the resected femur, patella, tibia, fibula, and foot, with their associated tendons and ligaments. Two different configurations were analysed: one with and one without the fibula, allowing the resulting stress state to be assessed and subsequently the contribution of the fibula to the torsional stiffness of the lower limb to be determined. Removal of the fibula results in increased stress on the remaining bones, particularly the femur and tibia, and on all tendons and ligaments. However, the extent of this increase varies between different structures. This information is critical for healthcare professionals dealing with injuries or surgery involving the fibula, as it highlights the need to consider how these procedures will affect overall lower limb function and load distribution. The fibula plays a crucial role in load distribution within the lower limb, reducing the load on the tibia and femur.^5^ Removal of the fibula requires other structures to compensate, resulting in increased stress.^21^ Over time, this can affect the performance and possibly the longevity of these tendons and ligaments. The effects on specific bones vary, with Tibia 5 and Femur 1 showing the most significant increases in stress without the fibula. Both models show an increase in stress with angular displacement, but the increase is more pronounced in the model without the fibula. There is a non-linear relation between the angular displacement and the stress; stress values increase more sharply as the angle increases. Increased stress on these structures can increase the risk of injury, particularly in high-impact activities such as sports or heavy manual labour. Understanding the stress pattern can help to prevent injuries, particularly to the femur and tibia, which carry the heaviest loads.^22^ It can also contribute to the development of effective rehabilitation strategies and the design of prostheses or orthoses that better mimic the natural stress distribution in the lower limb. Most of the other studies were based on compression loading, with a particular focus on the contribution of the fibula to the tibia, and most of the studies only applied loading to the proximal tibia, excluding the knee joint or femur. In the present study, the torsional stiffness resulting from compression and subsequent rotation of the femur on the tibia was obtained with and without an intact fibula. This was intended to be a baseline study for future studies investigating consequences of fibula resection on the torsional stability of the lower extremity. Özkan^9^ studied stress distribution in an FE model subjected to axial and torsional loading, consisting of the talus, fibula and tibia. The results confirm excellent agreement with our results, with values of 45 MPa in the tibia for the healthy model and 50 MPa in a fractured fibula model. The study by Kumar et al.^10^ showed that fibular plate fixation improved initial rotational stability after distal tibial fracture compared to tibial intramedullary nailing alone. It was found that there was no discernible difference in rotational structural stiffness between the two groups of specimens, those that had undergone plate fixation and those that had not. The fibula contributes to the formation of the ankle joint, providing lateral stability and distributing forces during weight-bearing activities. The proximal fibular joint forms a biomechanically efficient system with the tibia and distal connection to the foot through ligaments and tendons to transmit forces and maintain stability. Studies such as that of Cheung et al.^20^ provide a detailed representation of the complex role of the fibula in lower limb biomechanics. The functional importance of the fibula can be revealed by understanding how injuries affect the fibula. Research by Rammelt et al.^23^ highlights how fibula fractures, which are commonly associated with ankle injuries, can disrupt the fibulo-talar ligaments and consequently compromise ankle stability. In addition, studies of rotational ankle injuries, such as that of Wong et al.,^24^ demonstrate how torsional forces can lead to fibular fractures and ligament injuries, and emphasise the role of the fibula in torsional stability. Following fibular fractures or ankle injuries, rehabilitation protocols often aim to restore normal biomechanics and optimise functional outcomes. A research by Docherty et al.^25^ highlights how deficits in ankle stability, often associated with fibular injuries, can affect functional performance and predispose individuals to recurrent injury. Effective rehabilitation strategies target proprioception, strength and neuromuscular control to improve ankle stability and restore torsional stiffness, as outlined by others.^26^^,^^27^ Advances in biomechanical modelling techniques and surgical approaches have highlighted the role of the fibula in maintaining lower limb stability. Studies using computer-aided design (CAD) models^28^, ^29^, ^30^ examine the stress and displacement patterns that occur in the femoral chain and highlight the critical interaction between the various anatomical components in maintaining structural integrity or even the phenomenon of stress shielding in the presence of varus or valgus knee deformity. This highlights how such malalignments affect the overall stability of the leg and the distribution of loads. Other papers^31^^,^^32^ report interesting evidence of the knee joint biomechanics, investigating the combined effects of the quadriceps and medial retinaculum on patellar instability during knee flexion due to an unbalanced medial retinaculum load effect, and illustrating how small changes in ligament tension can lead to significant biomechanical consequences. Several studies^33^, ^34^, ^35^, ^36^ have compared different surgical fixation methods for fibular fractures to determine their effect on ankle biomechanics and torsional stability. Such research guides the development of surgical techniques aimed at preserving fibular anatomy and optimising postoperative outcomes. Collectively, these papers highlight the importance of maintaining the anatomical integrity of the fibula and other lower limb structures to ensure optimal biomechanical function. They also highlight the need for precise surgical techniques and tailored rehabilitation protocols to maintain or restore the natural load distribution and stability of the lower extremity. Longitudinal studies tracking the outcomes of fibula fractures highlight the complex interaction between the fibula and tibia, particularly in the presence of fractures or implants. This relationship is critical to understanding and improving surgical and rehabilitative strategies for lower limb injuries. McConnell et al.^37^ conducted a pivotal study of supination external rotation fibular fractures and emphasised the role of stress testing to understand the fracture mechanics and subsequent effects on the fibula and tibia. Their work supports the need for a comprehensive approach to the diagnosis and treatment of fibula fractures to ensure proper alignment and function of the entire lower limb. Other studies investigate the biomechanical interactions between the fibula and tibia^38^ focusing on implants that can facilitate the healing process, by providing optimal stress distribution across the fracture site. This approach not only promotes bone healing, but also maintains the mechanical integrity of the fibula-tibia complex. Other studies^39^^,^^40^ show how different implant designs can influence the distribution of stress and strain within the tibia and surrounding structures, including the fibula. This comparison is essential in others to select the most appropriate surgical implants that support the tibia but also maintain the functional relationship with the fibula. Another research^41^ investigates the tibio-talar contact stress through experimental and numerical studies that provide insight into how stress is distributed across the ankle joint. Experimental and numerical studies that provide insight into how stress is distributed across the ankle. This research is critical to understanding in what way changes to the fibula, due to fracture or surgery, can affect the overall biomechanics of the lower limb, particularly the loading of the tibia and ankle joint. Taken together, these studies highlight the critical interdependence of the fibula and tibia in maintaining stability and function of the lower limb. They highlight the significance of comprehensive biomechanical assessments and tailored surgical approaches to ensure optimal outcomes for patients with fibular fractures or tibial implants. Understanding the relationship between these bones, particularly in the context of fractures or implants, is essential to improve surgical techniques, enhance rehabilitation protocols and ultimately ensure better long-term patient outcomes.

In this study, the FE model contains several simplifications that may affect the accuracy of the results. The bones are modelled as materials and based on averaged anatomical data, which does not fully capture the true nature of human bone tissue. The material properties assigned to the fibula and other components are derived from literature values, which can vary significantly according to the source and specific conditions (e.g., age, gender, health status), potentially leading to differences in simulated torsional stiffness. The boundary conditions applied, such as constraints and loads, are idealised and may not perfectly replicate physiological conditions in vivo. For example, the load distribution and muscle forces acting on the lower extremity during torsion are complex and dynamic, whereas the model uses static conditions. The interactions between the different anatomical structures are simplified and may influence the overall mechanical behaviour of the lower extremity during torsion. The results of the FE model are validated against available experimental data, but there is inherent uncertainty in both the experimental measurements and the predictive capabilities of the model. The ability to fully verify the accuracy of the model in predicting real-world behaviour is limited by the lack of comprehensive in vivo validation data.

## 5 Conclusions

Although smaller than the tibia, the fibula plays a fundamental role in maintaining the structural integrity and biomechanical function of the lower extremity. It acts as a lateral strut, contributing to the stability of the ankle and knee joints. In addition, the fibula acts as an attachment site for several muscles and ligaments that are essential for coordinating complex movements and providing stability during dynamic activities. This anatomical configuration ensures that the fibula exerts a significant influence on the torsional stiffness of the lower limb, resisting rotational forces and maintaining optimal alignment. When considering post-operative rehabilitation, it is important to consider the contribution of the fibula to torsional stiffness to help the patient achieve effective recovery. Targeted physiotherapy exercises to strengthen the muscles and ligaments associated with the fibula can improve stability and reduce the risk of re-injury. Understanding the biomechanical role of the fibula enables clinicians to develop personalised rehabilitation protocols that facilitate optimal healing and functional restoration, adapted to the specific needs of the patient. While the study provides insight into fibula role in lower extremity torsional stiffness, the clinical relevance of the results obtained must be interpreted with caution.

## Ethical consideration

Ethical considerations are not relevant to this study. This is because it is an experimental study involving finite element analysis and this study does not involve human subjects.

## Funding

This research did not receive any specific grant from funding agencies in the public, commercial or not-for-profit sectors.

## CRediT authorship contribution statement

Angelo Alito and Vincenzo Filardi: Conceptualization; Elvira Mantineo, Andrea Vitali: Investigation; Angelo Alito: Supervision; Antongiulio Bruschetta, Andrea Vitali, Elvira Maria Mantineo: Writing - original draft; and Angelo Alito, Vincenzo Filardi: Writing - review & editing.

## Declaration of competing interest

The authors declared no potential conflict of interest.
